# Application of dental pulp stem cells for bone regeneration

**DOI:** 10.3389/fmed.2024.1339573

**Published:** 2024-02-29

**Authors:** Ye Liu, Wei Xiong, Junyi Li, Huixian Feng, Shuili Jing, Yonghao Liu, Heng Zhou, Duan Li, Dehao Fu, Chun Xu, Yan He, Qingsong Ye

**Affiliations:** ^1^Center of Regenerative Medicine, Department of Stomatology Renmin Hospital of Wuhan University, Wuhan, China; ^2^Department of Orthopaedics, Shanghai Sixth People's Hospital Affiliated to Shanghai Jiao Tong University School of Medicine, Shanghai, China; ^3^Sydney Dental School, Faculty of Medicine and Health, University of Sydney, Sydney, NSW, Australia; ^4^Institute of Regenerative and Translational Medicine, Tianyou Hospital of Wuhan University of Science and Technology, Wuhan, China; ^5^Department of Oral and Maxillofacial Surgery, Massachusetts General Hospital, Harvard Medical School, Boston, MA, United States

**Keywords:** bone defect, cell therapy, cell-free therapy, dental pulp stem cells, bone regeneration

## Abstract

Bone defects resulting from severe trauma, tumors, inflammation, and other factors are increasingly prevalent. Stem cell-based therapies have emerged as a promising alternative. Dental pulp stem cells (DPSCs), sourced from dental pulp, have garnered significant attention owing to their ready accessibility and minimal collection-associated risks. Ongoing investigations into DPSCs have revealed their potential to undergo osteogenic differentiation and their capacity to secrete a diverse array of ontogenetic components, such as extracellular vesicles and cell lysates. This comprehensive review article aims to provide an in-depth analysis of DPSCs and their secretory components, emphasizing extraction techniques and utilization while elucidating the intricate mechanisms governing bone regeneration. Furthermore, we explore the merits and demerits of cell and cell-free therapeutic modalities, as well as discuss the potential prospects, opportunities, and inherent challenges associated with DPSC therapy and cell-free therapies in the context of bone regeneration.

## 1 Introduction

Bone defects resulting from severe trauma, tumors, inflammation, and other factors have become increasingly prevalent (1). Bone undergoes remodeling, growth, and development and have remarkable regenerative capabilities following injury (2). Bone defect of critical size exceeds the regenerative ability of ones’ leading to impaired healing and nonunion.

Ideal bone grafts should be osteoconductive, osteoinductive, biocompatible, mechanically compatible, and potentially degradable. To treat critical size bone defect, autologous bone grafting remains the gold standard in current practice (3), but is subject to several limitations, such as low availability and collection risk.

Stem cell-based therapies have garnered increasing attention in bone regeneration. Mesenchymal stem cells (MSCs), initially isolated by Friedenstein from bone marrow in 1976 (4), have been found to differentiate into various lineages, including adipocytes, chondrocytes, and osteoblasts. MSC possess immunomodulatory, anti-inflammatory, anti-apoptotic, and paracrine abilities (5). Dental pulp stem cells (DPSCs) are a type of MSC derived from dental pulp that exhibit MSC-like properties and have exceptional capabilities in nerve and bone regeneration (6), secreting various active components, including extracellular vesicles, growth factors, cytokines, and extracellular matrix (7). These cell-free components derived from diverse procedures and amenable to various formulations, such as cell lysates, extracellular vesicles, conditioned media. Several studies have shown that cell-free components of MSCs’ could offer superior therapeutic effect over MSCs treating diseases. In contrast to the substantial body of research on MSCs, DPSC-based regenerative medicine and tissue engineering applications is limited but fast growing, especially in the context of their cell-free therapeutic modalities designed for bone regeneration.

In this review, we provide a detailed overview of the latest progress about the research update on DPSCs in bone regeneration. We believe this review will serve as a guidance for researchers and practitioners to establish a clear understanding on the research update of DPSCs in bone regeneration.

## 2 Characteristics of bone regeneration

### 2.1 General properties of bone

Bone matrix mainly consists of the inorganic component, hydroxyapatite, and the organic component, type I collagen, which is a calcified intercellular matrix (8). These components constitute the infrastructure of the mechanical properties of bone, e.g., tensile and compressive strength. Four primary cell populations present in bone tissue are osteoprogenitor cells, osteoblasts, osteoclasts, and osteocytes (9). Osteocytes are predominantly located within the bone matrix, while the other three cell types are found at the edge of bone tissue. The maintenance of bone homeostasis hinges on the dynamic balance between the osteoclast and osteoblast.

Osteoblasts are mainly involved in the development of new bone and undergo three differentiation stages: osteoprogenitor cells, preosteoblasts, and osteoblasts. Osteoblasts are derived from MSCs through multiple differentiation pathways. Initially, in osteoblast cell lines, MSCs differentiate into osteoprogenitor cells (10). The expression of Sox family transcription factors marks the primary differentiation of osteoblasts. Sox4 and Sox11 (SOXC group) promote the survival of osteoprogenitor cells (11), while Sox9 indicates differentiation toward chondrocytes (12). Osteoblasts could transform into osteocytes, which interact with nerves, blood vessels, and tissue fluid to form osteons that provide structural support for the skeleton.

In addition, bone morphogenetic proteins (BMPs) also play a critical role in bone generation and repair (13), attracting the aggregation of preosteoblasts at the injured site and differentiating them into osteoblasts (14). Recombinant human bone morphogenetic protein-2 (rhBMP-2) was approved by the FDA in 2002 for use in anterior lumbar interbody fusion. However, complications have been identified, such as excessive osteogenesis, osteolysis, inflammation, edema, and carcinogenicity.

**Figure 1:**
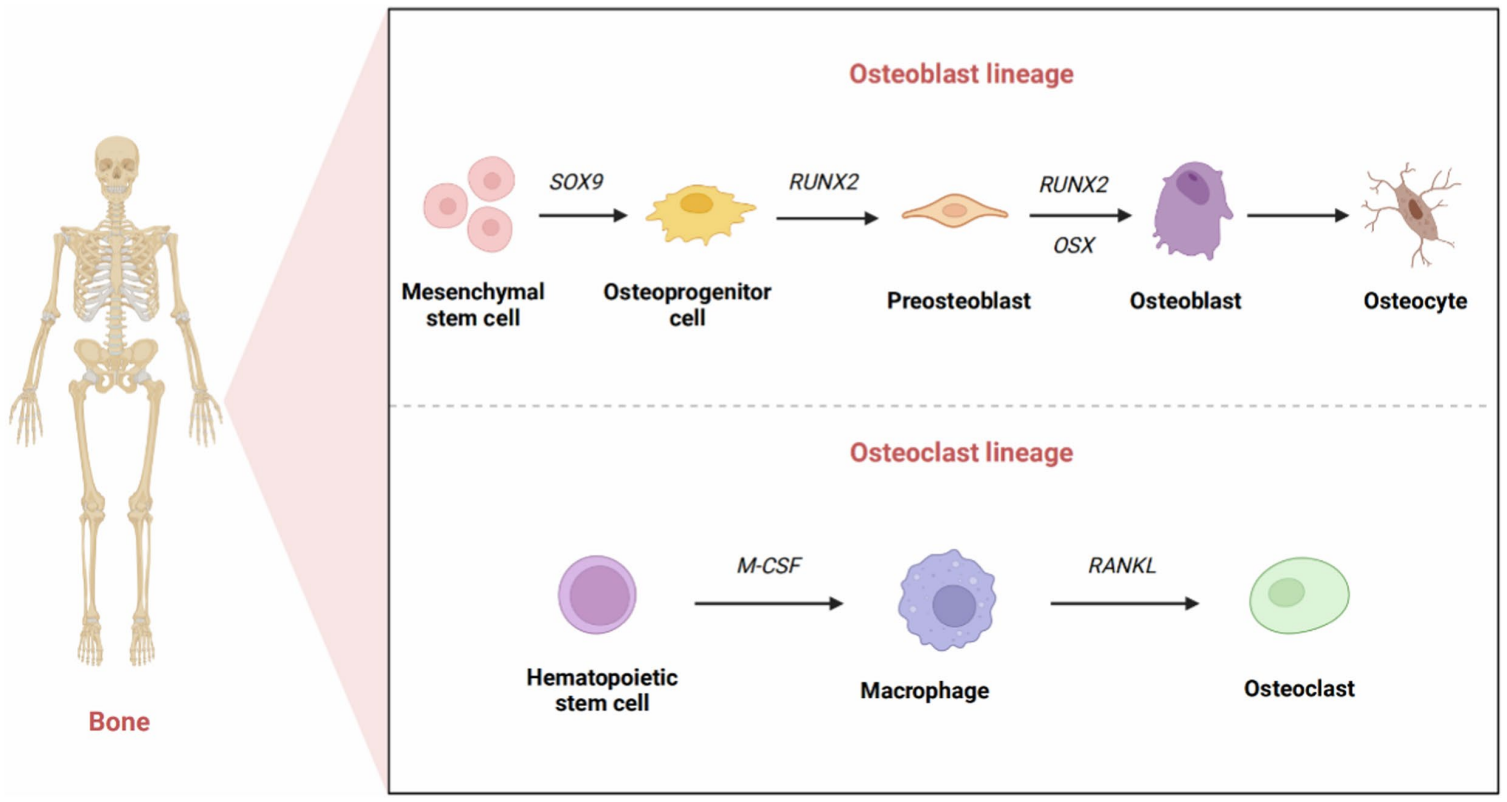
The development of osteoblasts and osteoclasts. The formation of mature osteoblasts has three differentiation stages: osteoprogenitor cells, preosteoblasts, and osteoblasts. Originally, the expression of Sox9 transcription factors marked the differentiation of osteoprogenitor cells. Runx2 signals the formation of preosteoblasts. The high expression of Osx reflects the differentiation of preosteoblasts into osteoblasts. Eventually, a portion of osteoblasts transformed into osteocytes that interact and combine with nerves, blood vessels, and tissue fluid to form osteons that provide structural support for the skeleton. Osteoclasts originate from hematopoietic stem cells. M-CSF promotes the proliferation of osteoclast precursors, and RANKL promotes the differentiation of mature osteoclasts.

Osteoclasts originate from hematopoietic stem cells, and their function is mainly to absorb and resorb bone. Hematopoietic stem cells can differentiate into osteoblasts, macrophages, dendritic cells, and other cell types (15). Macrophage colony-stimulating factor (M-CSF) and receptor activator of NF-κB ligand (RANKL), which can be produced by stromal cells, osteoblasts, and immune system cells, are essential in the differentiation and survival of osteoclasts (16). Interestingly, they are highly expressed in osteoblasts (17). M-CSF binds to the CSF-1 receptor to activate downstream signaling and further upregulate RANK expression, thereby promoting the differentiation of hematopoietic stem cells into osteoblast precursor cells (18). When activated by RANKL-RANK signaling, osteoclast precursor cells differentiate, fuse, and interact with osteoblasts to become mature osteoclasts (19). Osteoprotegerin (OPG) is also involved in this process. OPG, produced mainly by osteoblasts, is a soluble RANKL decoy receptor that inhibits osteoclast formation and bone resorption by preventing RANKL-RANK receptor interactions (20). M-CSF can stimulate cell survival signaling through activation of thymoma virus proto-oncogene 1 (commonly known as AKT) via phosphatidylinositol 3 kinase (PI3K), but it most notably activates extracellular signal-regulated kinase (ERK) via growth factor receptor binding protein 2 [Grb-2; (21)]. All these factors can indirectly induce osteoblast precursor cells to differentiate into mature osteoblasts (Figure 1).

The growth and development of human bone involve a continuous process of bone remodeling. In this process, osteoclasts absorb old bone matrix, and osteoblasts deposit sedimentary new bone matrix (8). However, bone regeneration after damage caused by trauma, tumors, or inflammation is a distinct regenerative process. This process commences with the onset of inflammation and the upregulation of proinflammatory factors at the site of damage, including tumor necrosis factor-α (TNF-α), interleukin-1 (IL-1), interleukin-6 (IL-6), and interleukin-11 (IL-11) among others (22). Subsequently, neutrophils and macrophages accumulate at injury site. The bone remodeling process occurs in four stages (19): recruitment and activation of osteoclasts at the site of injury; resorption of broken bone matrix by mature osteoclasts; differentiation of osteoprogenitor cells into mature osteoblasts; and aggregation of osteoblasts to form new bone matrix and mineralization. These stages are distinct and overlap with each other.

### 2.2 Pathways associated with bone regeneration

**Figure 2:**
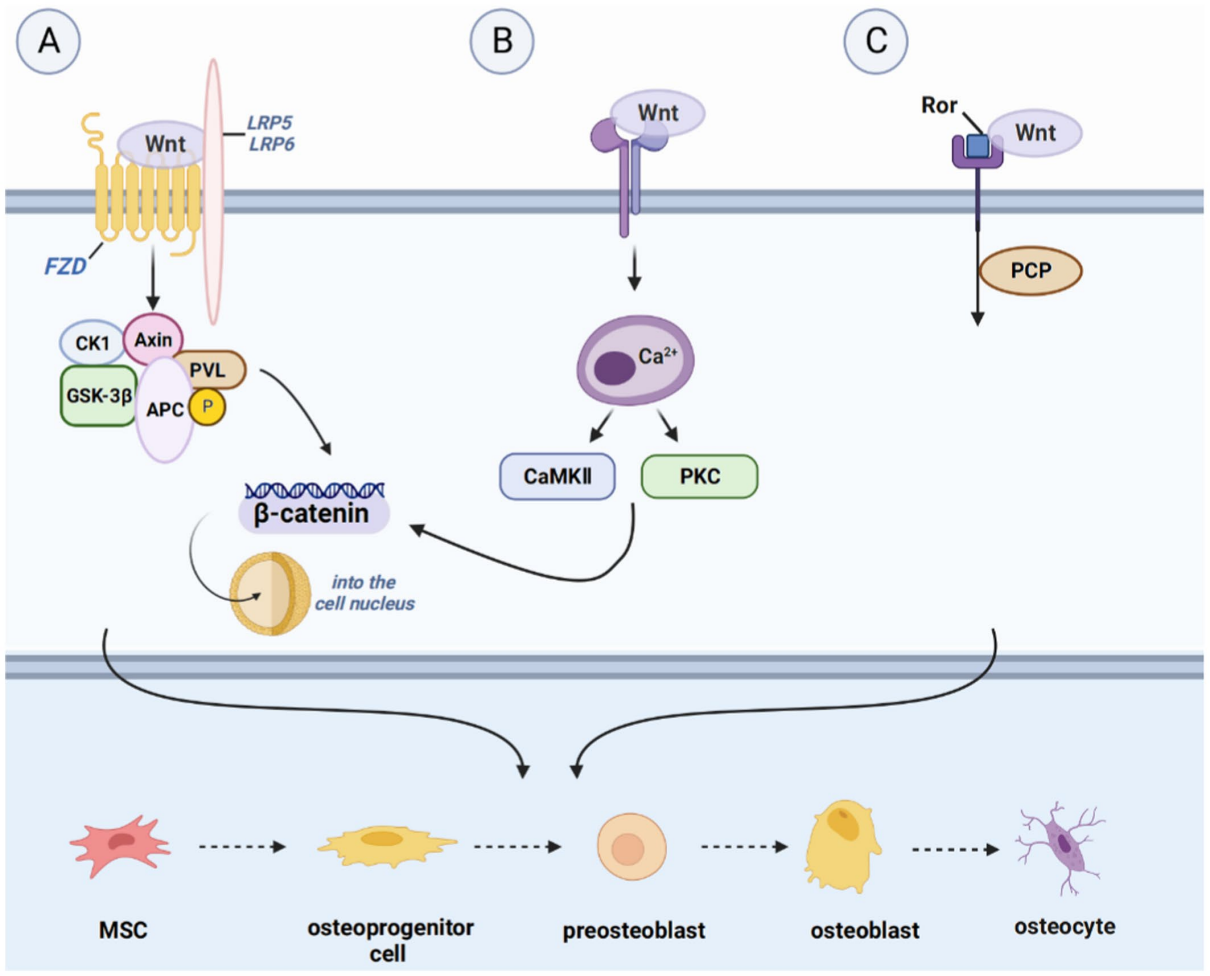
Introduction of pathways related to bone regeneration, like WNT signaling pathway. **(A)** IHH binds to Smoothened homolog (SMO) to activate GLI2 activator (GLI2A) and to prevent the cleavage of GLI3-to-GLI3 repressor (GLI3R), thus leading to the formation of osteocytes. **(B)** Hydrolysis of Notch proteins is achieved by Notch binding to JAG or DLL, followed by Notch intracellular structural domain (NCID) binding to EGF repeats present in the ligand to affect the expression of downstream target genes, including the split hairy enhancer (Hes) and Hes associated with the YRPW motif (Hey). Hes1 and Hey1 prevent osteoblast differentiation and maturation and promote bone resorption by inhibiting Runx2 activity, where the expression of Hes1 appears to be a key determinant of bone mass. **(C)** The FGF pathway activation begins with FGF binding to FGFR, with subsequent phosphorylation of tyrosine residues in the intracellular domain of FGFR, recruitment of various substrates, and activation of downstream pathways. This ultimately leads to cell proliferation, differentiation, and apoptosis.

**Figure 3:**
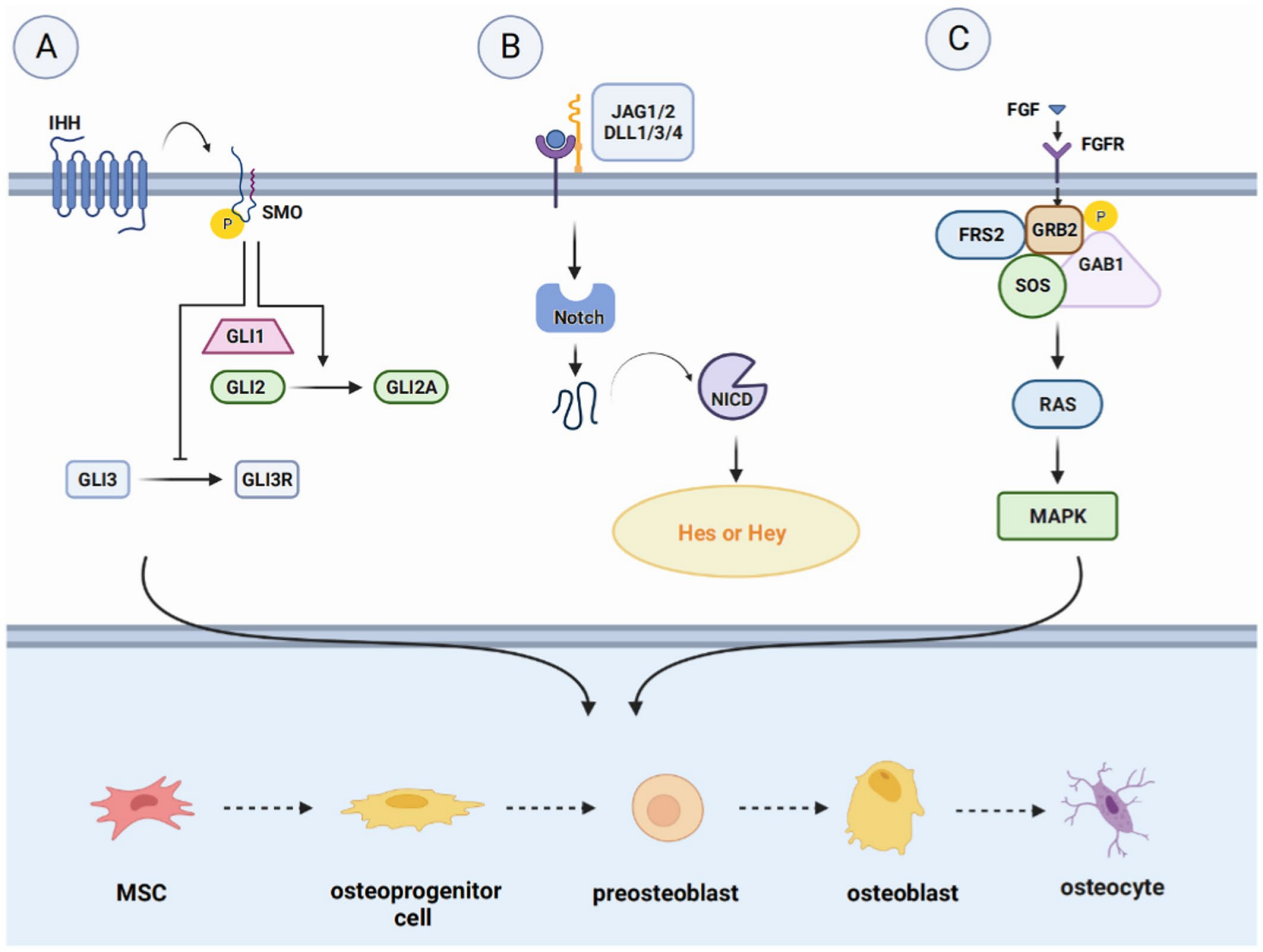
Introduction of pathways related to bone regeneration, like Hedgehog signaling pathway, Notch signaling pathway and FGF signaling pathway. **(A)** The WNT signaling pathway, divided into the WNT/β-catenin pathway, the WNT/Ca^2+^-dependent pathway, and the WNT/planar cell polarity (PCP) pathway. Upon binding ligands such as Wnt1 to the FZD receptor and LRP5/6 complex, Axin down-regulates and inactivates GSK-3β, inducing the accumulation of β-catenin in the cytoplasm and translocation to the nucleus to induce the expression of target genes. **(B)** In the Wnt/Ca^2+^ pathway, the increase in intracellular Ca^2+^ concentration activates CaMK II and protein kinase C (PKC) and facilitates the translocation of β-catenin to the nucleus. **(C)** Wnt activates WNT/PCP signaling through tyrosine kinase-like orphan receptor (Ror) proteins, promoting the formation of osteocytes and activation of downstream signaling pathways.

**Figure 4:**
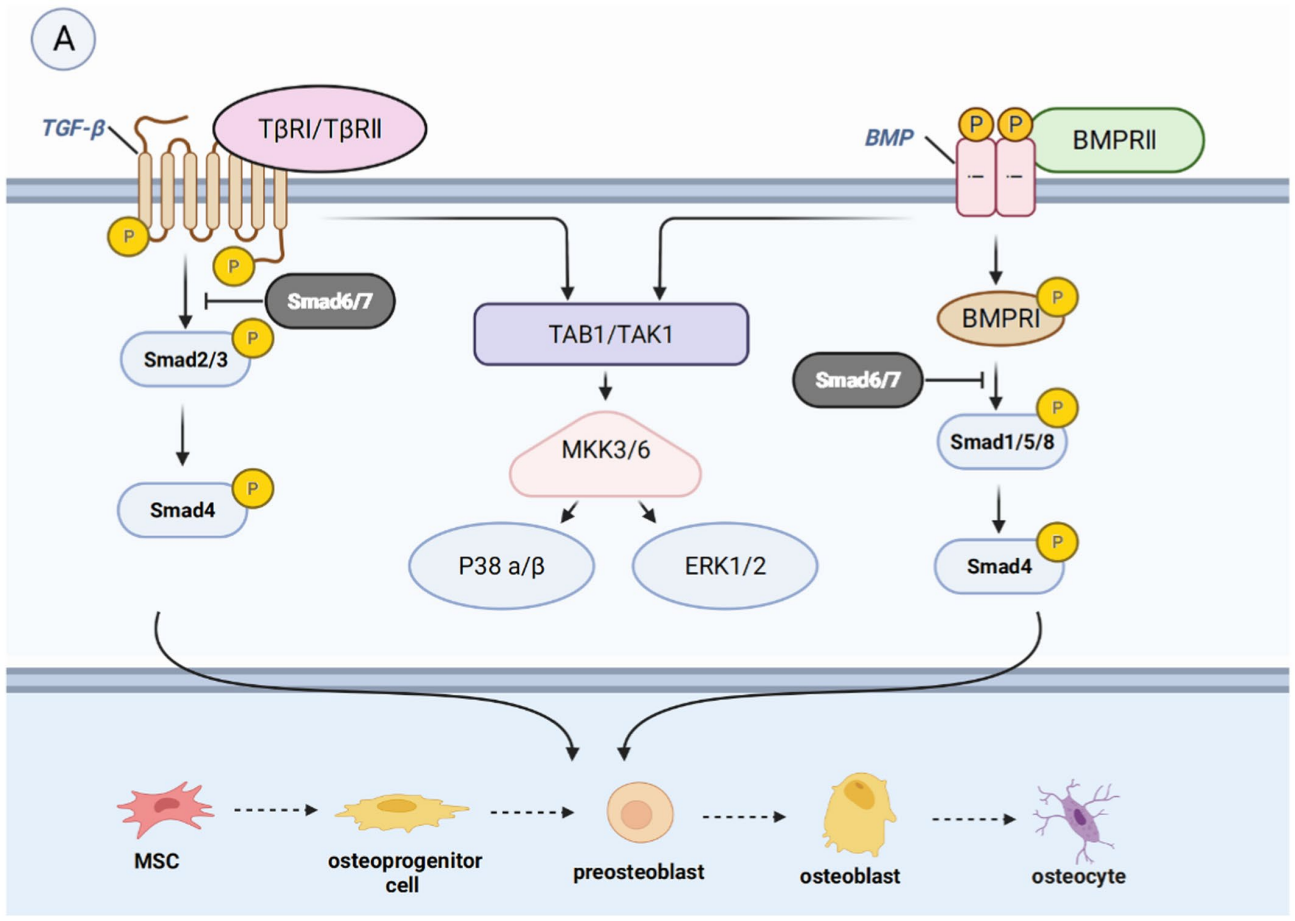
Introduction of pathways related to bone regeneration, like BMP/TGF-βsignaling pathway. **(A)** Binding of BMP leads to the phosphorylation of Smad1, Smad5, or Smad8. The binding of TGF-β leads to the phosphorylation of Smad2 or Smad3. They form a complex with Smad4, which then moves the nucleus to control gene expression and enable the transformation of mature osteoblasts. They can also activate non-smad-dependent pathways, including PI3K/AKT, TAK1, and MAPK signaling pathways, which are cascades of signaling events.

**Figure 5:**
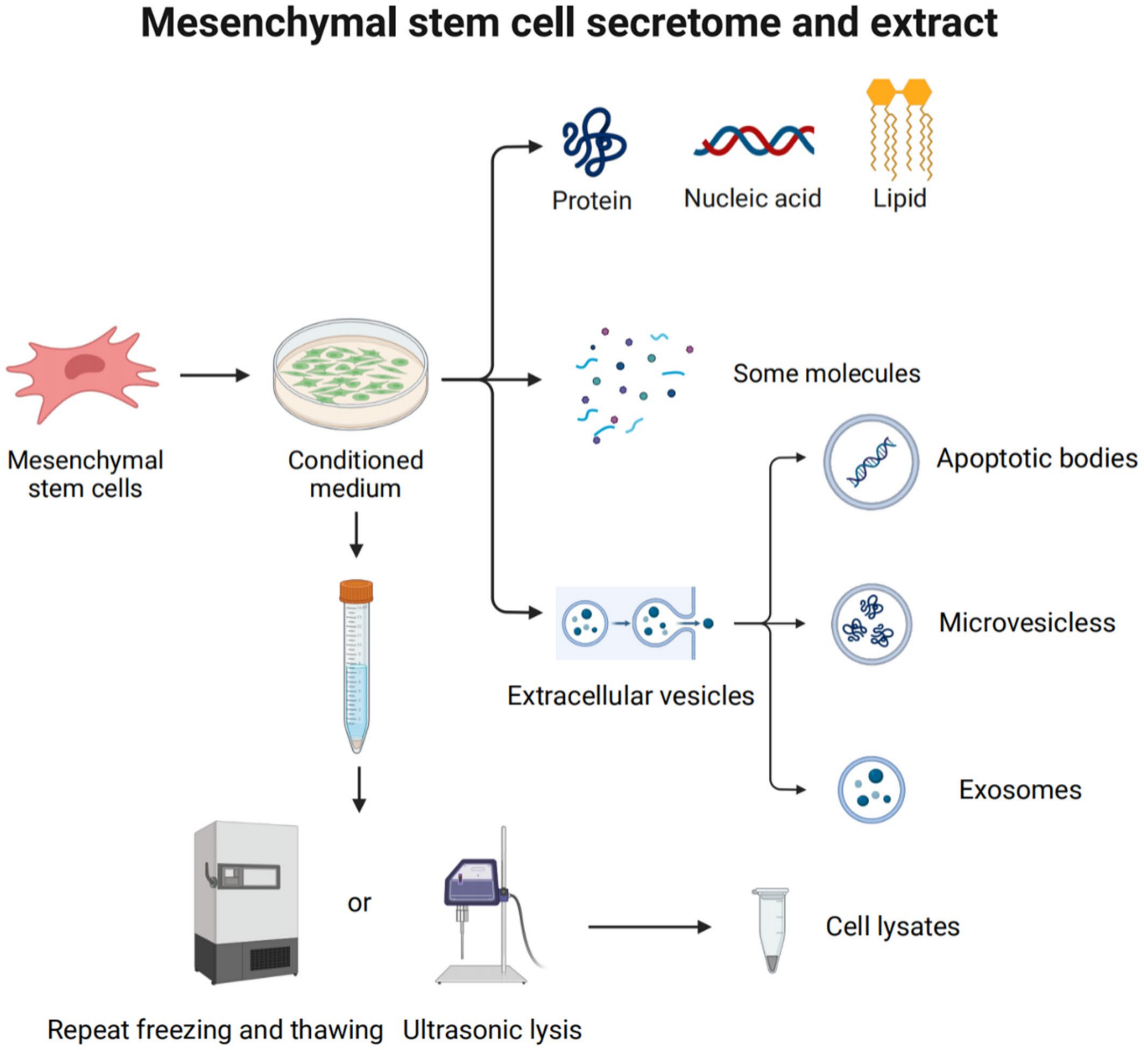
Mesenchymal stem cell secretome and extract. The MSC secretome, or CM, plays a major role in the use of MSCs to treat diseases. It is a variety of molecules and extracellular vehicles (EVs) secreted by MSCs into the extracellular space. These molecules include soluble proteins (such as growth factors, chemokines, enzymes, adhesion molecules, hormones, antimicrobial peptides, etc.), free nucleic acids, lipids, etc. EVs can be classified into apoptotic bodies (1–5 μm), microvesicles (250–400 nm), and exosomes (30–150 nm), according to their size and origin. Cell lysate is obtained by lysing cells through ultrasonolysis or freeze–thaw technology.

The mechanisms underlying bone regeneration have been extensively investigated, leading to the identification of various signaling pathways involved in this process (23). These pathways, such as the Hedgehog signaling (Hh) pathway, Notch signaling pathway, WNT signaling pathway, BMP/TGF-β and MAPK signaling pathway, IGF signaling pathway, and other signaling pathways (FGF signaling pathway, PI3K/Akt/mTOR signaling pathway, et al.), interact with each other to co-regulate bone regeneration (24) (Figures 2–5).

#### 2.2.1 Hedgehog signaling pathway

The differentiation of osteoblast lineages is regulated by multiple signaling pathways, with the Hh pathway being of critical importance in bone formation. Shh is a major morphogenetic factor in limb formation, regulating patterns and growth during early limb development. Takebe et al. found that Runx2 and Osx co-localized Shh and Gli1 on the surface of bone matrix and chondrocytes 7 days after fracture in SD rats, suggesting that Shh is involved in intramembranous ossification and endochondral ossification during fracture healing (25). On the other hand, Ihh is involved in several aspects of cartilage endosteal bone development. It is upregulated during the early stages of bone regeneration, expressed in prehypertrophic and hypertrophic chondrocytes, and indirectly regulates osteoblast differentiation by controlling cartilage development.

The Gli family is an important transcription factor involved in the Hh pathway, which includes Gli1, Gli2, and Gli3 (25). As a transcriptional repressor, Gli3 appears to play a dominant role in the control of chondrocyte proliferation and hypertrophy, while Gli2 activation may have a more prominent role in angiogenesis (26). The absence of these transcription factors can lead to abnormal skeletal development or postnatal death in mice (27). Gli1 acts synergistically with Gli2 and Gli3, and there is an inhibitory effect of Gli3 inhibitors on osteoblast differentiation, which is partially mediated through inhibition of Gli1 (28).

#### 2.2.2 Notch signaling pathway

The Notch signaling pathway plays a crucial role in regulating cell proliferation and differentiation in various tissues and organs, including skeletal development, bone metabolism, and regeneration (29). Four Notch receptors have been identified so far; they are Notch1-4 and have been identified in humans and mice. Studies have demonstrated that Notch1 overexpression leads to decreased expression of alkaline phosphatase transcripts, type I collagen, osteocalcin, and other proteins associated with bone regeneration during the differentiation of ST-2 stromal cells into osteoblasts. Additionally, Notch2 selective inhibition has been shown to hinder RANKL-induced osteoclast genes during osteoclast differentiation, while ectopic expression of Notch2 enhances NFATc1 promoter activity and promotes osteoclastogenesis (30). Notch3 activation in prostate cancer bone metastases has been shown to induce and secrete matrix metalloproteinase-3 (MMP-3), which subsequently inhibits osteoclast differentiation and enhances osteoblast proliferation (31). Pulsed electromagnetic field (PEMF) treatment has been shown to activate Notch pathways, stimulate all osteogenic markers, and increase the expression of Hey1, Dll4, and Notch4 in osteogenic media (32). This suggests that Notch4 plays a positive regulatory role in Notch signaling. It is worth noting that the stimulation or inhibition of Notch signaling in osteoblasts and osteoprogenitor cells may depend on the cellular environment, the differentiation status of the cell, or the developmental stage of bone formation (33).

Delta-like protein-1/3/4 and Jagged1/2 are five membrane-bound ligands that have been identified as activators of Notch signaling (34). Mechanistically, the binding of Notch to Jagged1/2 or Delta-like protein-1/3/4 leads to hydrolytic cleavage of Notch proteins, allowing the Notch intracellular structural domain (NCID) to bind to EGF repeats present in the ligand and affect the expression of downstream target genes. These genes include the hairy enhancer (Hes) and the Hes associated with the YRPW motif (Hey). Hes1 and Hey1 inhibit Runx2 activity, thereby preventing osteoblast differentiation and maturation and promoting bone resorption (35). Notably, the expression of Hes1 appears to be a key determinant of bone mass (36). Shen et al. have reported that in osteoblasts, Hes-1 enhances 1,25-Dihydroxyvitamin D3 (1, 25-(OH)2-D3)-induced osteopontin transcription and that this enhancement is inhibited by inhibitors of Runx2 (37).

#### 2.2.3 WNT signaling pathway

The WNT signaling pathway is a complex and versatile pathway that is present in all major systems. WNT signaling pathway, divided into the WNT/β-catenin pathway and two non-classical pathways, the WNT/Ca^2+^-dependent pathway and the WNT/planar cell polarity (PCP) pathway (38). Binding of extracellular WNT ligands to the Frizzled family of transmembrane receptors as well as lipoprotein receptor-related protein-5 (LRP5) and lipoprotein receptor-related protein-6 (LRP6) co-receptors activate the classical pathway (39). Wnt ligands are a class of secretive proteins that act through autocrine or paracrine mechanisms, and 19 different Wnt ligands are present in the human body. Wnt1, Wnt2b, Wnt3, Wnt7a, and Wnt8 are primarily involved in the WNT/β-catenin pathway.

Under normal conditions, cytoplasmic β-catenin is phosphorylated by a complex of glycogen synthase-3β (GSK-3β), adenomatous polyposis coli, and Axin and subsequently rapidly degraded by the ubiqui-tin-proteasome system. However, upon Wnt stimulation, Axin down-regulates and inactivates GSK-3β, leading to the accumulation of β-catenin in the cytoplasm, which translocates to the nucleus and induces target gene epistasis via TCF/LEF1 and CBP. *In vitro* studies have indicated that Wnt ligands promote the differentiation of MSCs into osteoblasts by activating the WNT/β-catenin pathway, thereby enhancing osteogenic bone mass, suggesting that the WNT/β-catenin pathway is essential in the osteogenic differentiation system (40).

The non-classical pathway includes the ligands Wnt4, Wnt5a, Wnt5b, Wnt7b, and Wnt11. Ligand binding to the receptor does not induce intracellular β-catenin accumulation, but it does participate in osteoblast and osteoclast differentiation. Maeda et al. found that Wnt5a activates WNT/PCP signaling through the tyrosine kinase-like orphan receptor (Ror) protein, thereby enhancing osteoclastogenesis and promoting bone resorption (41). In the Wnt/Ca^2+^ pathway, an increase in intracellular Ca^2+^ concentration leads to the activation of calmodulin-dependent protein kinase II (CaMK II) and protein kinase C (PKC), which in turn facilitates β-catenin translocation to the nucleus (42). Kuhl et al. showed that CaMK II and PKC activated by the Wnt/Ca^2+^ pathway block the Wnt/β-catenin pathway upstream of β-catenin and phosphorylate the intracellular protein (43). However, its downstream mechanisms are unclear.

#### 2.2.4 BMP/TGF-β signaling pathway

In most of the studies, it is believed that the BMP/TGF-β signaling pathway is crucial to bone biogenesis. The transcriptional growth factor beta (TGFβ) is synthesized by osteoblasts and embedded in the mineralized matrix. It is one of the most abundant cytokines present in the bone matrix, and it is more abundant than BMP *in vivo*. The BMP family constitutes the largest subfamily of the TGFβ superfamily. BMP is a cytokine essential for fetal tissue development and fracture repair and is considered a key factor in the lineage of MSCs to bone progenitor cells (44). The major intracellular mediators of the BMP/TGF-β signaling pathway are Smad molecules. Nine Smads molecules have been identified and defined, classified as receptor-regulated Smads (R-Smads), co-mediator Smads (Co-Smads), and inhibitory Smads (I-Smads). R-Smads consist of Smad1/2/3/5/8/9, among which Smad2/3 is mainly involved in the TGF-β signaling pathway, while Smad1/5/8/9 is mainly involved in the BMP signaling pathway. Co-Smad, also known as Smad4, is involved in the two pathways (45). I-Smad, which is divided into Smad6/7, exerts a negative regulatory effect on the TGF-β signaling pathway. In the typical Smad-dependent TGF-β or BMP signaling pathway (46), TGF-β binds to type II and type I TGF-β receptors, or BMP binds to BMP receptor II to form a heterotetrameric receptor complex. This complex activates phosphorylated R-Smads, which forms a complex with Smad4 and is subsequently recruited to the nucleus (47, 48). In the nucleus, the complex promotes the expression of osteogenic-related genes. In the presence of I-Smads, R-Smads cannot be phosphorylated to form complexes with Co-Smads, leading to the inhibition of this signaling pathway (45).

The non-Smad-dependent pathways include TAK1, MAPKs, and PI3K/AKT signaling pathways (49), which constitute cascades of signaling events. For example, phosphorylated TAK1 initiates the MKK-p38 MAPK or MKK-ERK1/2 signaling cascade by recruiting TAB1 (50, 51). Subsequently, Runx2, Dlx5, and Osx are phosphorylated to promote their transcriptional activity (52). However, some studies have demonstrated that these pathways may inhibit the osteogenic effect, indicating a dual role (53).

#### 2.2.5 Other signaling pathways

In recent years, there has been growing evidence indicating that the fibroblast growth factor (FGF) signaling pathway holds promise as a key regulator of bone regeneration. Studies have shown that FGF stimulates the proliferation and differentiation of MSCs and osteoblasts, contributing to bone formation (54). For instance, Nakajima et al. demonstrated that the injection of basic fibroblast growth factor (bFGF) into a rat model of fracture healing accelerates the rate of fracture healing and promotes bone regeneration (55).

The regulation of the PI3K/Akt/mTOR pathway in pathways that promote bone repair has not been extensively studied. Inhibiting the PI3K/Akt/mTOR pathway can lead to osteoclast formation; activating the pathway promotes cell differentiation toward osteoblasts (56). In a study by Peng et al., Akt1 and Akt2 gene-deficient mice showed severe skeletal muscle atrophy and impaired skeletal development (57).

### 2.3 Cross-talk between these pathways

Bone development and regeneration involve a complex interplay of various signaling pathways that coordinate and crosstalk with each other (49). Despite the distinct roles of these pathways, they function cooperatively to ensure proper bone formation and repair. The interaction between the TGF-β/BMP pathway and the Wnt pathway is critical for early development and tissue stabilization (58). TGF-β can prevent hyperphosphate-induced osteogenesis by inhibiting the Wnt/β-catenin pathway and reducing nuclear translocation of Smad 1/5/8 in the Smad-dependent BMP signaling pathway (59). Furthermore, Wnt3a inhibits Smad1 phosphorylation via GSK3 and stabilizes Smad1 (60). At Ser-204 and Ser-208 (61), GSK3 mediates Smad3 phosphorylation and inhibits its activity. Axin promotes GSK3β-mediated Smad3 phosphorylation at Thr66, leading to Smad3 ubiquitination and degradation (62). TGF-β regulates Hes-1 transcription in a Notch-dependent manner. The intracellular domain of Notch1 (NICD) can interact with Smad3, enhancing cascade signaling (63).

Although numerous pathways not listed here are also involved in bone regeneration and development, the mechanisms underlying skeletal development and repair are primarily a result of cross-talk between these pathways.

## 3 DPSCs therapeutic potential in bone regeneration

Stem cell-based therapies have been a major area of focus in the fields of tissue engineering and regenerative medicine. MSCs have been isolated from a variety of human tissues, including bone marrow, peripheral blood, umbilical cord synovial fluid, dental pulp, adipose tissue, and endometrial tissue (64). MSCs are characterized by their immunomodulatory properties, multi-directional differentiation potential, and high self-renewal capacity. These cells have been demonstrated to participate in diverse processes such as immune regulation (65), neuroprotection, anti-inflammatory, anti-fibrotic, anti-oxidant, and angiogenic processes (66). They also demonstrate advantages in the treatment of bone regeneration, degenerative diseases, diabetes, wound healing, and other areas (67). While theoretically, MSCs can be isolated from any human tissue, there are limitations due to the availability of the source tissue, the invasive nature of the isolation procedure, and the different characteristics of the donor. Obtaining bone marrow mesenchymal stem cells (BMMSCs), for example, may present risks of pain, bleeding, or infection, making it challenging and ethically controversial (68). Therefore, it is crucial to select a suitable cell source, assess the difficulty of obtaining samples, and consider the rejection of allogeneic cell transplantation.

### 3.1 The unique characteristics of DPSCs

DPSCs have emerged as a prospective cell source for stem cell-based therapies that can be readily available from third molars, deciduous teeth, or permanent teeth (69). Third molars are usually discarded as medical waste, and two sets of teeth germinate during a person’s lifetime, indicating that DPSCs are abundant and easily accessible. DPSCs have MSC-like properties, such as multidirectional differentiation and the ability to self-renew. Compared with other sources of MSCs or progenitor cells, DPSCs are not only easy to obtain but also have low immunogenicity and avoid ethical concerns. More importantly, DPSCs can be collected without injury to the donor or invasive surgical procedures (70).

Because of their origin in the cranial neural crest lineage, DPSCs have significant neural differentiation potential, making them suitable in treatment of neurological problems (71). However, their advantages in bone regeneration are also unquestionable. DPSCs exhibit higher fibroblast colony-forming units and proliferation rates, similar gene expression profiles of mineralization-related genes, and differential osteogenic, paracrine, and immunomodulatory capacity as compared to BMMSCs (72). The osteoblasts in the craniofacial region are also derived from neural crest cells, which are derived from neural ectoderm. During the embryonic period, the neural ectoderm develops into the tissues of the craniofacial region, including the facial bones, the skull, and the dentin of the teeth (23). Therefore, DPSCs have great osteogenic potential (73).

### 3.2 Bone regeneration and immunomodulatory properties of DPSCs

In the past few years, the majority of scholars have shifted their focus to inducing bone regeneration through the transplantation of BMMSCs, adipose-derived mesenchymal stem cells (ADSCs), and umbilical cord-derived mesenchymal stem cells (UCMSCs). Firstly, bone marrow mesenchymal stem cells are heterogenous cell populations located in the medullary stroma of bone marrow, originating from the early development of the mesoderm and outer layer. They are the earliest-discovered and extracted type of mesenchymal stem cells. BMMSCs possess a strong ability to differentiate into bone cells and can be used for repairing bone defects (74). By implanting BMMSCs into the defective area, it is possible to promote bone tissue growth and reconstruction (75). However, due to their low proliferation capacity, high collection risks, and the painful collection process, researchers have been searching for alternative sources of mesenchymal stem cells (76). Subsequently, adipose stem cells and umbilical cord mesenchymal stem cells have been applied to treat bone defects. ADSCs, compared to other stem cells, exhibit excellent proliferation and differentiation potential (77), capable of promoting tissue regeneration and functional recovery. UCMSCs possess strong immune regulation and anti-inflammatory abilities (78), are able to alleviate inflammatory reactions and promote tissue recovery, but have a relatively weak osteogenic capability and are associated with ethical controversies (79).

Like other types of stem cells, DPSCs exert their therapeutic effects primarily through paracrine signaling. Specifically, DPSCs secrete a wide range of bioactive molecules, including regulatory factors, growth factors, cytokines, and signal peptides (80). DPSCs also release secretory proteomes, including exosomes, microvesicles, apoptotic vesicles, and other extracellular factors (81), which act in the body to provide a suitable environment for immune regulation and anti-apoptosis, maintaining overall body homeostasis. Importantly, DPSCs exhibit low immunogenicity (82), and the expression of histocompatibility complex class II antigen does not exist on DPSCs. These unique properties have led to the development of various therapeutic applications for DPSCs in treating neurological, circulatory, diabetic, liver, eye, immune, and oral diseases (83).

The regenerative potential of dental pulp stem cells (DPSCs) in the field of craniofacial bone repair is not to be underestimated. Gaus et al. conducted a study using data from the Gene Expression Omnibus database, which revealed 16 overlapping differentially expressed miRNAs and shared regulators associated with bone differentiation between DPSCs and BMMSCs, suggesting a common genetic and epigenetic mechanism for bone differentiation in both cell types (84). An *in vitro* study has demonstrated that DPSCs exhibit superior osteogenic potential compared to other MSCs, including BMMSCs, gingival mesenchymal stem cells (GMSCs), and adipose-derived stem cells (ADSCs), as evidenced by various assays such as fluorescence-activated cell sorting, flow cytometry, quantitative polymerase chain reaction for osteogenic gene expression, alizarin red staining, and micro-computed tomography analysis (85). However, other conflicting results have been reported (72, 86–94), which may be attributed to differences in MSCs donors and isolation techniques. Table 1 summarizes the inconsistent findings on the osteogenic potential of DPSCs. Nevertheless, DPSCs show advantages in terms of proliferation rates, cell utilization, and cell numbers compared to BMMSCs (90, 92). These advantages are reflected in Table 2. DPSCs also exhibit remarkable immunomodulatory, paracrine, anti-apoptotic, and angiogenic properties, which contribute to bone differentiation and reduce inflammation (91, 96, 98, 101). Anderson et al. showed that DPSCs reduce inflammation and induce M2 polarization in bone marrow cells (102). The same conclusion was drawn *in vivo*. In a rabbit cranial defect model, inoculation of BMSC or DPSC in a Bio-Oss stent and implantation into a 6-mm cranial defect promoted bone regeneration and improved osteogenesis-related protein expression at the defect site, and the bone regeneration efficacy of the two cells was shown to be compatible (94). In a temporomandibular joint arthritis rat model, local injection of DPSCs was found to alleviate inflammation and pain in the joint cavity, promote bone regeneration, and inhibit the STAT1 signaling pathway (103).

**Table 1:** Comparison of osteogenic potential between DPSCs and BMMSCs.

Author	Cell resource	Proliferation capacity	Mineralization potential	Conclusion
Pierdome-nico et al. (86)	Human	DPSCs>BMMSCs	BMMSCs = DPSCs	Compared with BM-MSCs, DP-MSCs exhibited a lower differentiation capacity and a superior proliferation pattern.
Stanko et al. (87)	Human	DPSCs>BMMSCs	BMMSCs > DPSCs	DPSCs were more proliferative than bone marrow BMMSCs.
Davies et al. (88)	Rat	BMMSCs > DPSCs	DPSCs > BMMSCs	The mineralization pattern of DPSC cultures differs from that of ADSCs and BMMSCs in that DPSC cultures lack well-defined mineralized nodules.
Isobe et al. (89)	Human	—	BMMSCs > DPSCs	The differentiation potential for osteogenesis and chondrogenesis was found to be higher in BMMSCs than in DPSCs.
Aghajani et al. (90)	Human	BMMSCs > DPSCs	BMMSCs > DPSCs	The osteogenic differentiation potential of DPSCs is lower than BMMSCs.
Zhang et al. (91)	Human	DPSCs > BMMSCs	BMMSCs > DPSCs	BMMSCs have better osteogenic capacity than DPSCs.
Kanemats-u et al. (92)	Human	DPSCs > BMMSCs	BMMSCs > DPSCs	BMMSCs are superior to DPSCs in osteogenic differentiation.
Kumar et al. (93)	Human	DPSCs > BMMSCs	DPSCs > BMMSCs	Dental stem cells (especially DPSC) have a higher osteogenic potential compared to BMSC.
Lee et al. (94)	Rabbit	BMMSCs = DPSCs	BMMSCs > DPSCs	The *in vitro* results have shown that the BMMSCs possess a higher osteogenic differentiation potential than the DPSCs.
Mohanram et al. (95)	Human	DPSCs > BMMSCs	DPSCs > BMMSCs	In conclusion, the hDPSCs have better osteogenic ability and higher proliferation rate.
Mohanram et al. (95)	Human	DPSCs > BMMSCs	DPSCs > BMMSCs	In conclusion, the hDPSCs have better osteogenic ability and higher proliferation rate.
Lyu et al. (72)	Rat	—	BMMSCs = DPSCs	Compared with rBMSC-CellSaic, rDPSC-CellSaic showed better ability to promote bone regeneration.

BMMSCs > DPSCs, BMMSCs is superior to DPSCs in one aspect. DPSCs > BMMSCs, DPSCs is superior to BMMSCs in one aspect. BMMSCs = DPSCs, BMMSC and DPSC showed no difference in one aspect. —, Not mentioned.

**Table 2:** Comparison of the immunomodulatory and other abilities of BMMSCs and DPSCs.

Cell resource	Immunomodulatory capacity	Paracrine capacity	Angiogenic ability	Anti-apoptotic ability	Author
Human	DPSCs > BMMSCs	—	—	—	Pierdome-nico et al. (86)
Porcine	DPSCs > BMMSCs	—	DPSCs > BMMSCs	—	Ishizaka et al. (96)
Human	DPSCs > BMMSCs	DPSCs > BMMSCs	DPSCs > BMMSCs	—	Mead et al. (97)
Porcine	—	DPSCs > BMMSCs	DPSCs > BMMSCs	DPSCs> BMMSCs	Hayashi et al. (98)
Human	—	—	—	DPSCs> BMMSCs	Zhang et al. (91)
Human	DPSCs > BMMSCs	DPSCs > BMMSCs	—	DPSCs> BMMSCs	Ji et al. (99)
Rat	DPSCs > BMMSCs	—	—	DPSCs> BMMSCs	Abbas et al. (100)
Human	DPSCs = BMMSCs	—	—	—	De et al. (101)

BMMSCs > DPSCs, BMMSCs is superior to DPSCs in one aspect. DPSCs > BMMSCs, DPSCs is superior to BMMSCs in one aspect. BMMSCs = DPSCs, BMMSC and DPSC showed no difference in one aspect. —, Not mentioned.

The ability of DPSCs to promote bone regeneration is mediated by various mechanisms. Ferutinin, a phytoestrogen, promotes osteogenic differentiation of the Wnt/β-catenin signaling pathway by activating H3K9 acetylation and H3K4 trimethylation in the promoter regions of Wnt3a and DVL3 (104). Similarly, adenosine A1 receptors have been shown to promote DPSCs osteogenesis through the Wnt/Dvl pathway, as evidenced by increased expression of the osteogenic markers RUNX-2 and ALP (105). AR-A014418, a glycogen synthase kinase 3β (GSK3β) inhibitor, promotes not only the proliferation and migration of DPSCs but also their osteogenic differentiation, which is achieved through the activation of the β-catenin/PI3K/Akt signaling pathway (106). DPSCs also inhibit osteoclasts by inactivating the AKT pathway through the secretion of OPG, as demonstrated by Kanji et al. (107). Melatonin affected the osteogenic differentiation ability of DPSCs through COX-2/NF-κB and p38/ERK MAPK signaling pathways, which was also verified in the rabbit calvarial defect model (108). In addition, some drugs, enzymes, hormones, or trace elements can induce DPSCs to express osteogenic-related genes through the BMP4/Smad pathway, the Erk1/2 pathway, the p38 MAPK pathway, and other pathways to promote bone regeneration and repair (109, 110). Notably, the formation and connectivity of blood vessels are crucial for osteogenesis (111), and DPSCs have been shown to possess a higher angiogenic potential than other MSCs (112).

## 4 Cell-free therapies based on DPSCs for bone regeneration

Cell-free therapy has emerged as a promising approach in regenerative medicine due to its potential to address some of the drawbacks and limitations linked to the use of MSCs. While the ability of DPSCs in osteogenesis is well established, there is growing evidence to suggest that the therapeutic effect of MSCs may be derived from their paracrine bioactive factors, which have a critical role in the regulation of cellular processes (67). Adverse reactions have been reported with the use of MSCs, either intravenously or topically, such as mild fever, headache, dizziness, venous obstruction, and adverse reflux (113). The survival time of MSCs after entering the body is short and is affected by the microenvironment. Furthermore, the long-term culture of MSCs may lead to a loss of phenotypic stability (80, 114). Consequently, attention has shifted toward safer and more practical cell-free therapies.

The MSC secretome, also known as conditioned medium, is a secretory molecule of MSCs that has a variety of biological activities, is actively involved in regulating cellular processes, and can be used for various therapeutic applications (115). These molecules include soluble proteins (such as growth factors, chemokines, enzymes, adhesion molecules, hormones, antimicrobial peptides, etc.), free nucleic acids, lipids, some vesicles, etc. (116). Many of these have immunomodulatory, anti-apoptotic, anti-fibrotic, and tissue-regenerative functions. Among them, extracellular vehicles (EVs) are considered important mediators of intracellular communication and are involved in the regulation of multiple signaling pathways (117). EVs can be classified into apoptotic bodies (1–5 μm), microvesicles (250–400 nm), and exosomes (30–150 nm), according to their size and origin (118). Apoptotic bodies, containing histone and DNA fragments, are the largest extracellular vesicles that split from cells during apoptosis. Microvesicles are produced by the plasma membrane budding and are rich in sphingomyelin and ceramides. Exosomes are budded from the interior of the multivesicular body, which fuses with the plasma membrane and releases exosomes outward, containing a series of evolutionarily conserved proteins like tetrameric proteins and heat shock proteins (119).

In summary, cell-free therapies in the form of the MSC secretome, or CM, could offer a safer and more practical alternative to cell therapy, with the potential for greater efficacy in the treatment of bone defects (73). The EVs within the secretome are particularly important in regulating cellular processes and communication and hold promise as therapeutic agents in their own right (67, 120).

### 4.1 Conditioned medium

MSCs are known to secrete a variety of substances, including numerous proteins, peptides, RNA, and lipid mediators, as well as an abundance of extracellular vesicles (EVs). Mesenchymal Stem Cell Conditioned Medium (MSC-CM) (121) is derived from the supernatant of cultured MSCs and is a mixture of hundreds to thousands of different enzymes, growth factors, cytokines, and proteins that can be concentrated, frozen, or even lyophilized without loss of activity. MSC-CM can be collected in a simple and efficient manner. Cells are inoculated in a culture dish and cultured with serum-containing medium until the density is approximately 70%, after which serum-free medium is added, and the culture continues for 48 h. The supernatant is then collected and filtered with a sterile 0.22 μm filter to remove cell debris and bacterial microorganisms. The final supernatant is referred to as conditioned medium for MSCs and can be stored at −80°C until use or further concentrated and stored at ultra-low temperatures (122).

MSC-CM from different sources has been identified to have diverse and beneficial effects on the receptor, including anti-inflammatory, immunomodulatory, and angiogenic effects (123). Huang et al. used periodontal membrane-derived stem cell-conditioned medium, concentrated 20-fold, to culture chondrocytes, synovial cells, and meniscus cells isolated from IL-1β-treated porcine knees. The results validated increased expression of anti-inflammatory factors as well as decreased expression of inflammatory factors, confirming the anti-inflammatory effect of MSC-CM (124). Gharaei et al. analyzed multiple pro- and anti-angiogenic factors in DPSC-conditioned medium (DPSC-CM), cultured with human umbilical vein endothelial cells (HUVEC). They reported that these factors affected cellular migration and proliferation, stimulated tubulogenesis and promoted angiogenesis such as the number of nodes, meshes, and total tubular length (125). Proteomic analysis has uncovered a total of 1,533 proteins in CM derived from ADSCs, BMMSCs, and DPSCs, which have regenerative potential in areas such as cell migration, angiogenesis, inflammatory response, ossification, and organ survival. Moreover, the expression of multiple cytokines associated with odontoblast differentiation and anti-inflammatory cytokines was significantly higher in DPSC-CM (126). Paschalidis et al. found that DPSC-CM enhanced the viability, migration, and mineralization potential of DPSCs and even counteracted TEGDMA-induced cytotoxicity (127). Through *in vivo* and *in vitro* studies, Fujio et al. showed that anti-inflammatory, angiogenesis, and osteogenic-related factors were more highly expressed in DPSC-CM under hypoxia and promoted osteogenesis and accelerated bone healing in mouse tibia (128). DPSC-CM also increases mineralization potential through TGF-β1 expression, thereby triggering new bone formation and improving osteoblast and chondrogenic markers (128). New blood vessels are particularly important in the bone regeneration process, allowing better reconnection of new bone to its own bone. Ishizaka et al. showed that *in vivo* DPSC-CM exhibited higher immunomodulatory capacity as well as higher angiogenic and anti-apoptotic capacity *in vivo* compared to BMMSCs-CM (96).

### 4.2 Extracellular vesicles

All mammalian cell types studied so far, including neuronal cells, endothelial cells, MSCs, and epithelial cells, have been found to release EVs. EVs can be found in all kinds of body fluids like saliva, synovial fluid, urine, and blood. While EVs were previously considered to be cellular waste, recent research has shown that they have a major role in regulating cellular signaling pathways in target cells, including tumor cell growth, cell migration, cell communication, and angiogenesis (129).

EVs are small membranous vesicles that are released into the extracellular matrix by cells via the plasma membrane during the budding process (117). EVs contain a variety of substances, including phosphatidylserine, cytoplasmic proteins, mRNA, miRNA, DNA, and other molecules. Exosomes (Exos) are one group of the smallest and most extensively studied MSC-derived EVs. The genesis of Exos occurs through the endocytic exocytosis pathway, with early endosomes maturing, extending to late endosomes, and budding inward at the late endosomal membrane of multivesicular bodies. After the fusion of these multivesicular bodies with the cell membranes, MSC-derived Exos are released into the extracellular environment. These Exos carry important signaling molecules and exert biological effects in target cells (67, 130).

Various methods are available for the isolation of EVs and Exos, including ultracentrifugation, density gradient centrifugation, exosome kits, ultrafiltration, immunoprecipitation, and acoustic nanofilters (131). Ultracentrifugation is the gold standard method for Exos extraction, which involves multiple rounds of centrifugation at varying speeds to collect Exos from cell culture supernatants (132, 133). Density gradient centrifugation, on the other hand, separates Exos with less contamination but is more complex and time-consuming (134). Recently, commercial kits based on polymer coprecipitation have been developed for Exos extraction, such as ExoQuick, which is simple to use and has a high yield but may contain impurities (135). While each method has its advantages and disadvantages, ultracentrifugation is considered to be the most reliable and efficient technique for Exos extraction. However, this method can be expensive, time-consuming, and require large sample volumes. In contrast, commercial kits are simpler and faster but may have a higher level of impurities. Density gradient centrifugation offers higher purity, but the preparation is more complicated and time-consuming, and Exos cannot be completely separated from proteins. Overall, the choice of isolation method should depend on the specific research needs and resources available.

Exos have emerged as promising candidates for bone defect regeneration (136, 137). Studies have shown that Exos can bind to scaffolds or growth factors and modulate both osteoblast and osteoclast functions (138). Exos have also been found to induce osteogenic differentiation and bone regeneration by increasing osteoblast differentiation-related miRNA expression and inhibiting Axin1-activated Wnt signaling (139). High-throughput miRNA sequencing has revealed that Exos secrete 41 miRNAs that are differentially expressed after osteogenesis induction. These miRNAs have been implicated in bone differentiation and development, including osteoclast differentiation, the PI3K-AKT signaling pathway, the MAPK signaling pathway, and the mTOR signaling pathway. Among these miRNAs, hsa-mir-328-3p and hsa-mir-2110 have been identified as potentially the most important osteogenic regulatory miRNAs in Exos (140). In addition, Wei et al. prepared exosome-treated titanium nanotubes to enhance the osteogenic potential of BMP2 via natural nanocarriers (141).

Moreover, exosomes from human exfoliated deciduous teeth (SHED-Exos) have been shown to stimulate BMMSCs to express more osteogenic-related genes, such as Runx2 and ALP. Surprisingly, they also down-regulate lipopolysaccharide (LPS)-induced expression of inflammation-related factors in BMMSCs.

Although less research has been conducted on the use of dental pulp stem cell-derived exosomes (DPSC-Exos) in bone regeneration, their advantages are evident. DPSC-Exos have been shown to regulate target cells by translocating mRNA, miRNA, proteins, and other molecules to receptor cells. Hu et al. analyzed the microRNA profile of DPSC-Exos and verified that miR-27a-5p of DPSC-Exos promotes odontogenic differentiation of DPSCs by downregulating LTBP1 (one of the suppressor molecules of TGFβ1 signaling) to regulate the TGFβ1/Smads signaling pathway and thus express more osteogenic-related genes (142). DPSC-Exos have also been proven to enhance the proliferation and migration of haplotype homo dental pulp cells and mouse osteoblasts, inhibit the formation of mouse osteoclasts, and effectively reduce bone loss caused by periodontitis (143). Combining DPSC-Exos and DPSCs with β-tricalcium phosphate, hydroxyapatite, or collagen in a rat skull defect model has been found to accelerate bone regeneration and promote more extensive angiogenesis at the defect site. Notably, DPSC-Exos and DPSCs were found to have almost identical effects at the site of bone defects (144). Although DPSC-Exos has been shown to have bone tissue repair effects, the mechanisms behind these effects are not fully understood.

### 4.3 Cell lysate

Cell lysate, a product of cell lysis, is a promising source of bioactive compounds for regenerative medicine and tissue engineering applications. Although the specific components of the cell lysate remain uncertain, it is known to contain several soluble nutrients, including growth factors, EVs, Exos, and other proteins. To obtain the cell lysate, cells are first cultured and then lysed using trypsin digestion, followed by centrifugation and resuspension in ultra-pure water, and finally subjected to ultrasound or freeze–thaw technology. At this stage, cell releases various proteins and soluble nutrients, including growth factors like EGF and IGF. It can be used in the same way as MSCs, CMs, EVs, Exos, etc. to treat diseases.

While there is limited literature on the utilization of cell lysate for treating bone defects, several studies have demonstrated its anti-inflammatory and regenerative properties in other applications. For example, Jiao et al. were surprised to find that MSC-conditioned medium (MSC-CM) or MSC lysate (MSC Ly) can substantially enhance IL-10 secretion by peripheral blood mononuclear cells (PBMCs) *in vitro*. Simultaneously, it can significantly increase serum IL-10 levels in two animal models and reproduce the effects of an MSC graft *in vivo* (145). Ward et al. used the classical hind-paw edema model to simulate temporomandibular joint osteoarthritis, after treatment with human umbilical perivascular mesenchymal cells and their lysates, there were significantly lower concentrations of myeloperoxidase and TNF-α at 48 h. Treated osteoarthritis demonstrated lower concentrations of leukocytes in the synovium compared to controls and histologic evidence in the peri-articular tissue of reduced inflammation (146). Similarly, lysates of olfactory mucosa tissue-derived mesenchymal stem cells (OM-MSCs), when cultured with LPS-stimulated normal human liver cells (LO-2), inhibited the inflammatory process and promoted the proliferation rate of LO-2. Similarly, in a mouse model of LPS-induced acute liver injury, OM-MSCs lysate treatment attenuated inflammation and reduced liver enzyme release, thus alleviating liver injury (147). Khubutiya et al. reported that a mixture of BMMSC-conditioned medium (BMMSC-CM) and MSCs lysate significantly enhanced liver regeneration and reduced injury in a mouse model of acetaminophen-induced acute liver injury (148). Moreover, the administration of filtrated adipose tissue-derived mesenchymal stem cell lysate for three consecutive days mitigated inflammation and inhibited cell apoptosis in a mouse model of acute colitis, leading to improved survival rates, reduced weight loss, and clinical signs (149). Erectile dysfunction (ED) remains a major complication after radical prostatectomy. Albersen et al. found that penile injection of both ADSC and ADSC-derived lysate can improve recovery of erectile function in a rat model of neurogenic erectile dysfunction (150). Our research team has found that the lysates of DPSCs can be used for anti-photoaging treatment, and ZIF-8, as a safe and effective protein delivery system, can improve the skin bioavailability of DPSC lysates, demonstrating significantly enhanced cell uptake and skin retention ability (151). The cell lysate extraction process is rapid, simple, and produces a diverse range of precipitated components with excellent efficacy, thus holding great potential for future applications in regenerative medicine and tissue engineering.

### 4.4 Advantages and limitations of cell-free therapy

Recent experimental and clinical studies have shown that the use of the MSC secretome is a highly successful therapeutic strategy. MSC-CM and MSC-derived EVs have demonstrated similar therapeutic efficacy to MSC transplantation in bone and cartilage defects (152). The replacement of cell therapy with cell-free therapy eliminates the undesirable side effects associated with live cell transplantation, including immune rejection, emboli formation, tumorigenicity, arrhythmias, calcified ossification, and disease transmission. The secretome can be stored at ultra-low temperatures, such as −80°C, for prolonged periods of time. Cryopreservation or freeze-drying does not compromise the effectiveness of the cell secretome. Whereas cell cryopreservation necessitates the use of potentially toxic cryopreservation agents and harsh cryopreservation temperatures. In emergency situations, the secretome can be rapidly thawed for immediate use. Lastly, following clinical translation, the cell secretome can be standardized in terms of dose and potency, akin to a clinical drug (153).

Despite all the benefits of cell-free therapy, there are some limitations to its application. For instance, there are currently no standardized protocols for producing large quantities of EVs or extracellular vesicles. Furthermore, soluble factors secreted by MSC have not been fully elucidated. And it is unclear whether the use of CM, EVs, or cell lysate is safe for treating various diseases. Most significantly, the legal regulations surrounding cell-free therapy have not yet been fully established, given the rapid evolution of this field. Cell-free therapy remains in its nascent stages, and additional research is required to fully understand its potential as a therapeutic strategy (154).

## 5 Conclusion and future prospects

Critical size bone defect is a difficult problem to solve in modern medicine. In recent years, tissue engineering and regenerative medicine have shown good results in using DPSCs to treat bone defects. Since the first isolation of DPSCs, DPSCs have been identified to promote proliferation, differentiation, immune regulation, anti-inflammatory, anti-apoptosis, and paracrine signaling. DPSCs demonstrate good therapeutic effects when applied to various systemic diseases. It is noteworthy that they also exhibit strong regenerative capabilities for bone defects.

Cell-free therapy as a new therapeutic approach was discovered in the fields of regenerative medicine and tissue engineering, utilizing. The secretome of DPSCs could effectively avoid the risks in using stem cells (155). In the future, DPSCs secretome or its combination with biomaterials would enhance bone regenerative (156).

In summary, the use of DPSCs as a treatment for bone defects has shown promise but is still in the pre-clinical stages. The emergence of cell-free therapies has opened up possibilities for rapid access to therapeutic solutions for DPSC application.

## Author contributions

YeL: Conceptualization, Writing – original draft, Writing – review & editing. WX: Conceptualization, Writing – original draft, Writing – review & editing. JL: Supervision, Writing – review & editing. HF: Supervision, Writing – review & editing. YoL: Writing – review & editing. HZ: Writing – review & editing. LD: Writing – review & editing. DF: Writing – review & editing. CX: Methodology, Supervision, Writing – review & editing. YH: Methodology, Supervision, Writing – review & editing. QY: Conceptualization, Supervision, Methodology, Writing – review & editing.
